# Case of Spasmodic Contraction of the Voluntary Muscles, Particularly Those of the Superior Extremities, Consequent on Inhalation of the Vapours of Burning Charcoal

**Published:** 1817-09

**Authors:** Shirley Palmer


### *Case of Spasmodic Contraction of the voluntary Muscles, particularly those of the superior Extremities, consequent on Inhalation of the Vapours of burning Charcoal.* By Shirley Palmer, M.D.

NOXIOUS consequences from long exposure to the vapours extricated by charcoal during its combustion, and even their fatal effects upon the animal œconomy, have too frequently been witnessed to require comment or description in this place. But the evident production, through their agency, of a train of spasmodic symptoms, formidable in character, and surviving, for years, the direct operation of the aërial poison upon the system is, if I mistake not, a fact without precedent, or at least hitherto unrecorded, in the history of our art. No similar example, gleaned either from reading or observation, presents itself to my recollection. If such fact then be as novel to others as it is to myself, a brief narrative of the principal circumstances connected with it, may not be unacceptable to the readers of this Journal.

One Sunday night, about eight years since, in the depth of severe winter, a poor family, at Nuneaton near Coventry, composed of five persons, retired to rest in a chamber, wherein, for the sake of warmth, a chaffing dish, filled with burning charcoal, had previously been placed. With a view of more perfectly excluding the air, a blanket was drawn across the window. But, most fortunately, the door communicating with the room below, had been left open.

The family consisted of a man and woman, both in middle life; a stout boy and girl, respectively aged eleven and eight; and a boy of four years, who had been subject, almost from his cradle, to frequent paroxysms of epilepsy.

On lying down in bed, the elder boy expressed pleasure at the comfortable sense of warmth with which he was affected; and sank into a sleep from which he never rose again. How the hours of midnight were passed by this ill-fated family, is not known. Towards day-break, the father was disturbed by the moanings of his eldest boy. But he felt himself too ill to investigate the cause of the suffering, or offer assistance to the lad. At length, however, after re-iterated attempts, he arose from his bed, and immediately fell completely senseless on the chamber floor.

From this situation he imagines himself to have been revived by the fresh air blowing on his face through the crevices of the floor. However this be, he, in a short time, recovered sufficiently to escape down stairs, open the door, and alarm the neighbours. The following were the consequences of this unfortunate accident.

The man and woman had suffered much less severely than the children; and hence soon recovered. But both declare that, from this period, they have never enjoyed health so comfortable as it had previously been.

Into the mouth of the elder boy, who, on the entrance of the neighbours, was found insensible and "rattling in the throat," a quantity of spirit was poured.* Neither this, nor the introduction of fresh air, served at all to arouse him from his lethargy. He died between the hours of nine and ten that morning.

The girl, who forms the subject of my history, had, till this time, been remarkably free from all complaint, except habitual constipation of the bowels. During the whole of the day which succeeded the eventful night, she continued so ill as to require medical attendance; and, towards evening, was first seized with the spasmodic affection, of which I shall presently give a description. This, with some intervals of exemption, uncertain both as to the period of their occurrence and their duration, had ever since tormented the girl, and disabled her for service or other regular employment. The attacks were sometimes protracted, with little intermission, during several weeks. Terror, fatigue, or other mental or corporeal agitation, served instantly to re-excite them, when absent. They were aggravated about the commencement of the menstrual process; and conspicuously, though not permanently, relieved by the uterine discharge. They frequently came on in the night; and severe pain was felt during their invasion.

The younger and epileptic boy continued very ill for some time after his removal from the fatal chamber. His fits, from that period, obviously assumed an aggravated character; and his trunk and limbs became frequently bent and horribly distorted by spasmodic contractions of the muscles, to which he had never before been subject.

* It was afterwards found that little, if any, of the fluid had been swallowed.

He died in the spring of 1816, during an epileptic paroxysm.

Such was the statement delivered to me by the girl herself, and corroborated by an intelligent relative who accompanied her to my house on one of my public mornings in February last. For the individual accuracy of the circumstances here detailed, I obviously cannot vouch; but the narrative bears upon its front the stamp of general correctness.

The physical characters of my patient were those of florid and robust health. Her countenance heavy, and inexpressive of intelligence or warmth; and her moral dispositions, I have reason to believe, accorded pretty closely with these physiognomical indications. She was now in her 17th year.

The spasmodic affection was more conspicuously marked in the hands than elsewhere. But progression was sometimes impeded by the contraction of her feet; and I, now and then, observed, during my examination, a transient convulsion of the facial muscles. The fingers of both hands were closely drawn together; the thumb across the palmar surface; and the whole in a state of moderate flexion on the fore-arm. Great force was required to raise the thumb to its natural situation, and, on the removal of such force, it instantly relapsed. The muscles of the arm felt unusually rigid: the countenance occasionally assumed an expression of considerable pain.

The pulse and temperature of the surface were natural; the tongue loaded; the epigastric region sore on pressure; the abdomen prominent.

Menstruation was stated by the girl to be scanty and irregular; and the constipation of the bowels singularly obstinate. She declared that no medicines, of which she had taken great quantities under the direction of different gentlemen, had been of service to her; and that the only relief which she had ever experienced from the "drawing of her hands," was obtained by immersion of them in hot grains. She had recently been confined by serious illness; but respecting the character or treatment of the malady, I could collect no precise information.

After duly reflecting on these circumstances, I concluded that the torpor of the intestinal canal had been the predisposing cause of the complaint, and that it had been excited into action by diminution of the energy of the brain, resulting from the influence of the deleterious charcoal vapours. To remove the intestinal obstinacy and congestion, and arouse the dormant activity of the cerebral organ, were then the obvious indications by which the curative plan should be conducted. How far these theoretical reasonings were correct I know not. That they led to the adoption of successful practice is sufficient for me, and may perhaps be regarded by others as something like a test of their accuracy.

*Treatment.*— Feb. 17th. *Large doses of aloes and submuriate of quicksilver, every other night; sulphate of magnesia in senna infusion every morning: moderate exercise; light and sparing diet.* By these means, continued during three weeks, the bowels were freely evacuated, and the spasmodic affection conspicuously relieved.

March 10th. *Alkalis; the lytta, afterwards combined with aloes and gentian; sulphate of magnesia continued.* Little progress made during almost six weeks. Towards the close of this period, the bowels again became inactive, and the spasmodic affection was aggravated.

April 21st. *Full purgative plan of February resumed with decided benefit,* but the bowels yet torpid in the intervals of the operation of the mercurial.

May 8th. *Aloes, with two grains of the submuriate, every night; sulphate of magnesia every morning.* Bowels not sufficiently evacuated.

May 12th. *Nightly dose of the submuriate increased to four grains.* In about a week, the mouth became sore. *Mercurials discontinued; sulphate of magnesia every morning.* Some traces of the spasmodic affection yet remaining. *Electric sparks drawn twice a week from the arms and cervical portion of the spine; subsequently, smart shocks passed through them.*

June 2d. Perfectly free from complaint. Dismissed with directions to keep the bowels regular by the occasional employment of aloes, and to apply again in the event of any recurrence of the spasms. Nothing since has been heard respecting her.

It is not improbable, that a slight return of the spasmodic paroxysms may be experienced. But, from the controul which we have hitherto been able to exercise over them, there can exist little doubt of eventual success by a vigorous resumption of, and due perseverance in a plan which, suggested by theory, has been sanctioned by experiment.

Although my hopes and confidence in electricity, as a mean extensively applicable to the treatment of diseases, have long been destroyed, I cannot but regard it as having constituted an important, and even essential, agent in the cure of the affection which I have just been describing. In conjunction with purgatives, it has proved conspicuously beneficial in a case of spasmodic contraction of the *sterno-cleido-mastoid* muscle, which has recently fallen under my observation.

Fatal accidents from burning charcoal in close rooms, are of frequent occurrence among the inferior classes of society; and medical men cannot too often profit by the opportunities which they possess, of warning the poor and ignorant around them of the dangers attendant on such a practice. Some years since, myself and several others were thus made violently ill by the inhalation of the carbonic acid and carburetted hydrogen gases, ere the source of mischief was detected. A sense of oppressive warmth, intense and vehement pain in the head, with constriction of the temples, drowsiness, confusion of intellect, indistinctness of vision, and depression of muscular strength, held a conspicuous rank in the train of attendant symptoms.*

With the following practical remark I shall close this communication. A long-continued exhibition of purgative medicines has been recommended by Dr. Hamilton, in many formidable diseases, to the invasion of which the human system is exposed; and, in my opinion, that enlightened physician has conferred a greater benefit on our science by the description and illustration of his simple practice, than all the theoretical writers of the past and present century united. Why then is not this practice more generally employed? Why so rarely successful? Because medical men lack vigour and perseverance in the application of it. A few half doses of calomel are exhibited; the evacuations are not inspected; the patient gets no better; the spectre debility darkens the mental eye of the irresolute practitioner; and the purgatives are discarded. But, for the sake of common sense and humanity, let us apply to this plan the rules and principles by which we are directed in the administration of other remedies. What reflecting man would order half a pint of warm

* The reader, who is desirous of more precise information respecting the symptoms of asphyxia from the vapours of burning charcoal, may consult an article on asphyxia in the first volume of the *Medico-Chirurgical Journal*, page 174; an excellent paper by Dr. Babington, in the first volume of the *Medico-Chirurgical Transactions;* or Larrey's *Memoires de Chirurgie Militaire,* tome iii. p. 13.

gruel to be squirted into the rectum, in a case of obstructed colon, when distension of the intestine by the injection of quarts, or even gallons of the fluid can alone dislodge the obstructing mass? Who would content himself with a solitary blood-letting of a few ounces, in active and incipient pneumonia; when, by the repeated abstraction of pounds, the patient can only be rescued from impending destruction? What would have been the fate of the purgative plan in the case which we have just been contemplating, had it not been vigorously resumed after ts first imperfect and ineffectual trial? I have seen a pan-full of indurated fæces expelled by one half-scruple dose of calomel, combined with aloes, after a notable perseverance for weeks in the frequent use of the "milder cathartics." I have given to a girl, affected with obstinate hysteria, and who had in vain swallowed packets, without number, of nervine, antispasmodic, and tonic trash, five grains of calomel, succeeded by large doses of jalap every day for five weeks; nor till near the close of that period was the state of the intestinal discharges sensibly improved, or the dawn of that relief visible, which shortly proved the prelude to her lasting restoration.

Tamworth, July 17, 1817.

